# Perceived physical activity barriers as predictors of nomophobia levels in sports science students

**DOI:** 10.3389/fpubh.2026.1726076

**Published:** 2026-03-30

**Authors:** Öznur Akpınar, Melek Kurşunel, Hande Baba Kaya, Nazlı Yanar Tunçel, Selahattin Akpınar

**Affiliations:** 1 Faculty of Sport Science, Karamanoğlu Mehmetbey University, Karaman, Türkiye; 2 Faculty of Sport Science, Düzce University, Düzce, Türkiye

**Keywords:** hierarchical model, physical activity barriers, psychology, smartphone, student

## Abstract

**Introduction:**

In today’s world, where smartphones have become an indispensable part of life, the existence of a department for treating smartphone addiction (nomophobia) in hospitals demonstrates that this is a public health issue. Nomophobia symptoms are exhibited by individuals of almost all ages, with young people being the least able to stay away from their phones. Against this backdrop, the present study aims to investigate whether perceived physical activity barriers among students at the Faculty of Sports Sciences have a predictive effect on their levels of nomophobia.

**Method:**

A total of 570 students enrolled in the Faculty of Sports Sciences participated in the study on a voluntary basis. The Üsküdar Nomophobia Scale and the Perceived Physical Activity Barriers Scale were employed. The data were analyzed using the Jamovi (2.3.28.0) statistical program with a 95% confidence interval and a significance level of 0.05.

**Results:**

A positive moderate correlation was found between participants’ nomophobia and physical activity barrier scores. The final regression model was statistically significant [*R* = 0.479, *R*^2^ = 0.230, *F* (6,563) = 27.955, *p* < 0.001], explaining 23% of the variance in nomophobia. Adding perceived physical activity barriers to the model significantly increased its explanatory power (Δ*R*^2^ = 0.213, *p* < 0.001). Perceived physical activity barriers were found to be the strongest predictor of nomophobia (*β* = 0.451, *p* < 0.001). Department (*p* = 0.006) and teaching type (*p* = 0.019) were also found to be significant predictors, whereas BMI, gender and academic year were not.

**Conclusion:**

The findings suggest that perceived barriers to physical activity are a significant factor in predicting nomophobia among university students. Reducing these barriers and promoting active lifestyles could encourage healthier technology usage behaviors and reduce excessive smartphone addiction.

## 1 Introduction

The rapid proliferation of smartphones has fundamentally transformed daily life, communication patterns and behavioral habits, particularly among young adults and university students (1). While smartphones offer advantages such as instant access to information, social connectivity and educational resources (2, 3), excessive use can lead to behavioral issues (4). One of the most prominent technology-related psychological phenomena associated with excessive smartphone use is nomophobia (no-mobile-phone-phobia), defined as the fear or anxiety experienced when unable to access or use a mobile phone. Deriving from the term ‘cell phone phobia’, nomophobia refers to the psychological discomfort associated with the fear of losing connection, running out of battery, or being unable to communicate via mobile devices (5). Recent research shows that nomophobia is particularly prevalent among university students due to their heavy use of smartphones for academic, social and recreational purposes (6, 7). The constant need to be connected can lead to compulsive control behaviors, emotional dependence on smartphones and increased anxiety when separated from mobile devices (8). Excessive smartphone use in academic settings has been linked to distraction, poorer academic performance, sleep disorders and a lack of engagement in behaviors that promote health (9, 10). As digital technologies become more integrated into daily routines, it is important to understand the psychosocial factors associated with nomophobia in order to combat it. In this context, physical activity emerges as a key element.

Physical activity is widely recognized as a critical determinant of physical and mental health in university students (11). Participation in regular physical activity contributes to improved cardiovascular fitness (12), enhanced psychological well-being and a reduced risk of chronic diseases (13). However, despite these well-documented benefits, many young adults fail to meet the recommended levels of physical activity. University students, in particular, often experience significant lifestyle changes, academic pressures and environmental constraints that can hinder their ability to participate in regular physical activity (14). Consequently, identifying the barriers preventing individuals from participating in physical activity has become a key focus of public health and behavioral research.

Perceived barriers to physical activity refer to personal, environmental and social factors that individuals believe prevent them from exercising regularly (15). Personal factors contributing to physical inactivity include insomnia (16), lifestyle, psychological disorders, and disability (17), as well as screen time (18), to name a few. Environmental factors include climate (19), culture, and income status (20). Barriers may include time constraints, academic workload, limited access to resources, lack of motivation, social constraints or psychological factors such as stress and fatigue. Studies have shown that individuals who perceive more barriers are significantly less likely to engage in regular physical activity (21). In recent years, researchers have begun to investigate the relationship between digital technology use and physical activity behaviors. In addition to the various causes of a sedentary lifestyle, barriers to physical activity, such as smartphone use, play an important role because they can prevent individuals from exercising.

Various studies have suggested that excessive smartphone use may contribute to a sedentary lifestyle by increasing screen time and reducing opportunities for physical activity (22, 23). Conversely, individuals who struggle to engage in physical activity may increasingly turn to smartphones for entertainment, social interaction or stress relief. This reciprocal relationship shows that psychological and behavioral factors related to technology use can influence lifestyle behaviors, such as participation in physical activity. Nomophobia, a psychological factor related to technology use, is also a factor that negatively affects lifestyle, and research in this area is growing rapidly. First appearing in the research literature in 2008, studies suggest that nomophobia has reached epidemic levels (24, 25). Studies support the finding that nomophobia is becoming more prevalent. A 2022 meta-analysis study, which included 20 countries, suggests that nomophobia is more prevalent among young people and young adults in non-Western countries and that its prevalence has increased since 2021.

Individuals experiencing nomophobia may exhibit undesirable behaviors in their daily lives. A recent bibliometric study examined nomophobia-related research focusing on sleep, learning and attention, coping strategies, academic performance, and health problems (26). Previous studies have shown that excessive smartphone use encourages sedentary behavior and consequently reduces individuals’ physical activity levels (27, 28). Furthermore, extensive research on nomophobia in young populations (5, 29) indicates that nomophobia is highly prevalent among university students (30, 31). A recent study found that problematic social media and internet use among university students was positively associated with psychological distress and nomophobia (32). Students on sports science programs are expected to have a greater awareness of and knowledge about physical activity, health promotion and active lifestyles. However, despite their academic background, previous research has shown that sports science students may encounter barriers that limit their participation in regular physical activity (33, 34). Similar to other student populations, academic workload, educational requirements, time management difficulties and technological distractions can affect lifestyle behaviors in students across disciplines. Therefore, examining behavioral and psychological factors such as nomophobia in this group could provide valuable insights. Furthermore, the growing digitalisation of education and social life could lead to increased smartphone dependence among students. Online learning platforms, communication with peers and instructors, and social networking applications have become an integral part of university life (35). While these technologies facilitate academic processes, they can also contribute to excessive smartphone use. Consequently, students who are more dependent on smartphones may be more susceptible to nomophobia.

Although the body of literature examining nomophobia and related factors is gradually expanding, some significant gaps remain. Previous studies have primarily focused on the relationship between nomophobia and physical activity levels, generally finding that people who use their smartphones more tend to be less active. However, relatively little attention has been given to the psychological and perceptual barriers that may prevent individuals from participating in physical activity. These perceived barriers represent individuals’ subjective evaluations of factors hindering participation in physical activity, and they may play a critical role in shaping the lifestyles of young adults. Furthermore, while nomophobia has been extensively studied among general university populations, few studies have been conducted on students in sports science faculties, despite them being expected to maintain active lifestyles due to their academic studies and sports participation. To our knowledge, no previous study has directly investigated whether perceived barriers to physical activity predict nomophobia levels in sports science students. Closing this knowledge gap could provide valuable insights into the interaction between lifestyle-related barriers and technology-related behavior patterns. The current study therefore aims to examine the relationship between perceived physical activity barriers and nomophobia levels, and to determine whether these barriers significantly predict different levels of nomophobia among sports sciences students. Accordingly, this study aims to contribute to existing literature by investigating the predictive value of perceived physical activity barriers in relation to nomophobia levels among sports science students. In this regard, the following research hypotheses have been formulated:

‘There is a relationship between nomophobia levels and perceived barriers to physical activity in sports science students’.

Perceived barriers to physical activity in sports science students positively predict their nomophobia levels.

The increase in perceived barriers to physical activity also raises the likelihood of experiencing nomophobia.

## 2 Materials and methods

### 2.1 Research model

**Figure 1:**
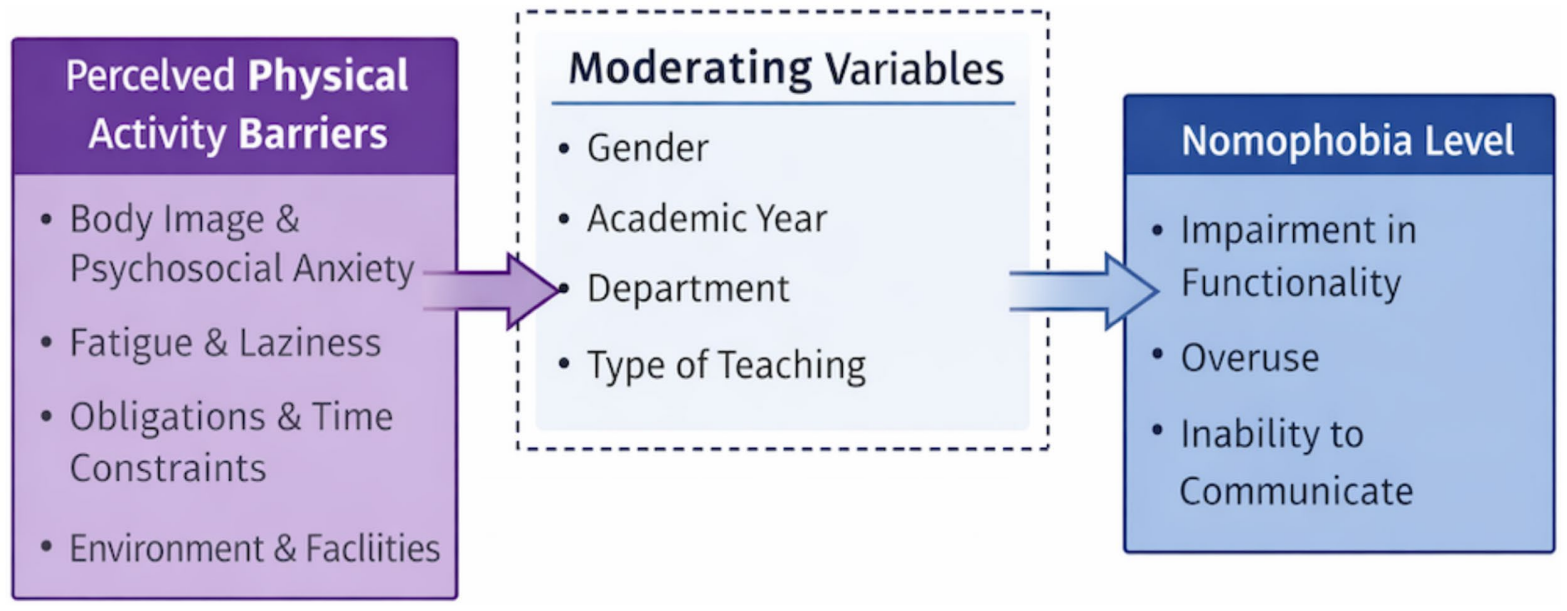
Conceptual model of the relationship between perceived physical activity barriers and nomophobia.

This study examines whether perceived barriers to physical activity among students at the Faculty of Sports Sciences predict their levels of nomophobia. To this end, a correlational survey model was employed. This model is used in quantitative research to reveal the relationship between two or more variables (36) (Figure 1).

### 2.2 Research group

An *a priori* statistical power analysis was conducted using G*Power (version 3.1) to determine whether the sample size was sufficient for the planned hierarchical logistic regression analysis. This analysis used a logistic regression test family, setting the two-tailed significance level (*α*) at 0.05 and the statistical power (1 − *β*) at 0.95. Based on previous studies examining behavioral and psychosocial predictors, a small-to-moderate effect size was assumed (odds ratio ≈ 1.5; corresponding *f*^2^ ≈ 0.10). Taking into account the three predictor variables (dimensions of perceived physical activity barriers) and the additional control variables included in the hierarchical model, the minimum required sample size to achieve sufficient statistical power was calculated to be around 138 participants. The current study included 570 participants, significantly exceeding the required minimum sample size. Therefore, the study possesses sufficient statistical power to detect significant relationships between perceived physical activity barriers and nomophobia levels in the hierarchical logistic regression model. Five hundred and seventy students studying at the Faculty of Sports Sciences at Karamanoğlu Mehmetbey University voluntarily participated in the study. The demographic characteristics of the participants are presented in Table 1.

**Table 1:** Demographic characteristics of the participants.

Variables	Group	*f*	%
Academic year	1. Grade	112	19.7
	2. Grade	228	40.0
	3. Grade	122	21.4
	4. Grade	108	18.9
Department	Coaching	289	50.7
	Sport management	191	33.5
	Physical education	90	15.8
Type of teaching	Normal teaching	417	73.2
	Night teaching (after 5 p.m.)	153	26.8
Nomophobia	Nope	216	37.9
	Low level	267	46.8
	Mid- level	83	14.6
	High level	4	0.7

The participants’ average age was (21.0 ± 2.9) years, average height was (173.4 ± 9.4) centimeter, and average body weight was (66.9 ± 13.0) kilogram.

### 2.3 Data collection procedure

The following tools were used in the study: the Demographic Information Survey, the Üsküdar Nomophobia Scale and the Perceived Physical Activity Barriers Scale.

### 2.4 Demographic information survey

This questionnaire, created by the researchers, collects information on participants’ gender, age, department, academic year and type of teaching.

### 2.5 Perceived barriers to physical activity scale

This scale, developed by in I-Maymi et al. (37), was used to identify perceived barriers to physical activity. Consisting of 17 items and three subscales (body image and psychosocial concerns; fatigue and laziness; obligations and time constraints; and environment and facilities), the scale uses a 5-point Likert scale. The lowest possible score is 17 and the highest is 85. The scale was adapted for the Turkish population in 2023 (38).

### 2.6 Üsküdar nomophobia survey

Participants’ level of nomophobia was determined using the Üsküdar Nomophobia Survey, developed by Yıldırım and Correia (39). This 25-item scale comprises three subscales (functional impairment, excessive use, and inability to communicate) and uses a 5-point Likert scale. The scale was adapted for Turkish society in 2022 (40). The scale can produce scores ranging from 25 to 125, with scores of 21–60 indicating low, 61–100 indicating moderate and 101–140 indicating high levels of nomophobia.

### 2.7 Data analysis

Before conducting the main analyses, the dataset was screened for missing values, outliers and violations of the normal distribution assumption. Descriptive statistics were calculated for all variables and reported as the mean (x̄), the standard deviation (SD), the frequency (n) and the percentage (%), where appropriate. The normality of the data distribution was examined using skewness and kurtosis values, histograms and the Kolmogorov–Smirnov test. According to the results of the normal distribution analysis, the skewness and kurtosis values were found to be within the desired range of ±1.5, indicating that the data followed a normal distribution (41). The internal consistency of the scales was evaluated using Cronbach’s alpha coefficients. Pearson correlation analysis was used to examine the relationships between nomophobia and perceived physical activity barriers and other variables for normally distributed variables. Hierarchical multiple regression analysis was performed to determine the predictive role of independent variables on nomophobia levels. Collinearity assumptions were evaluated using variance inflation factors (VIFs) and tolerance values. All analyses were performed using the Jamovi statistical program (version 2.3.21.0) at a 95% confidence interval and a significance level of 0.05.

## 3 Results

The means and normality analysis results for the participants’ scores on the Nomophobia and Perceived Barriers to Physical Activity Questionnaire are presented in Table 2.

**Table 2:** Mean, normality analysis and results of the data.

Scales	Sub-Dimensions	*N*	X¯	Sd	Skewness	Kurtosis	Cronbach’s α
Nomophobia	Impairment in functionality	570	19.3	7.4	0.7	−0.2	0.89
	Overuse	570	19.8	7.1	0.3	−0.3	0.89
	Inability to communicate	570	16.8	6.5	0.3	−0.5	0.89
	Total scale	570	55.9	17.9	0.2	−0.4	0.94
Physical activity barriers	Body image and psychosocial anxiety	570	2.7	0.4	0.5	−0.7	0.86
	Fatigue and laziness	570	3.7	0.3	0.1	−0.4	0.78
	Obligations and time constraints	570	3.6	0.7	0.2	−0.8	0.65
	Environment and facilities	570	3.2	0.8	0.2	−0.9	0.74
	Total scale	570	3.3	0.1	0.3	−0.2	0.91

Examination of Table 2 reveals that the participants’ mean total nomophobia score is (55.9 ± 17.9). These values suggest that participants exhibit low to moderate levels of nomophobia (39). In the Perceived Barriers to Physical Activity Questionnaire, participants achieved an average score of (3.6 ± 2.2) for the ‘Obligations and Time Constraints’ sub-dimension. Examination of the scale and its sub-dimensions reveals that all sub-dimensions and the total scale scores exceed 0.70, except for the ‘Obligations and Time Constraints’ sub-dimension in the perceived physical activity barriers scale. The Cronbach’s alpha coefficient for this subscale was found to be 0.65, indicating a moderate level of internal consistency, which is acceptable given the limited number of items in the subscale. While reliability coefficients are generally recommended to be 0.70 or above, values between 0.60 and 0.70 may be considered acceptable, particularly in exploratory studies or when a subscale contains a limited number of items (42). The results of the relational analysis of the participants are presented in Table 3.

**Table 3:** Correlation results for body mass index, nomophobia and physical activity barriers scores.

Variables	Body mass index	Nomophobia	Physical activity barriers
Body mass index	1.0		
Nomophobia	0.01	1.0	
Physical activity barriers	0.07	0.46***	1.0

*p < 0.05.

Examining Table 3 revealed a medium positive correlation between nomophobia scores and physical activity barriers scores (*r* = 0.46, *p* < 0.05). However, no significant relationship was found between body mass index and nomophobia (*r* = 0.01) or between body mass index and physical activity barriers (*r* = 0.07) (*p* > 0.05). Hierarchical multiple regression analysis was then used to determine whether scores on the perceived physical activity barriers scale could predict nomophobia scores (see Table 4). Before performing the regression analysis, the model assumptions were examined. Multicollinearity was evaluated using the variance inflation factor (VIF) and tolerance values. The results showed that the VIF values ranged from 1.015 to 1.259 and the tolerance values from 0.795 to 0.985, indicating that there was no multicollinearity problem among the predictors. The independence of the residuals was also evaluated using the Durbin–Watson test. The Durbin–Watson statistic was found to be 1.813, indicating that there is no autocorrelation in the residuals (43).

**Table 4:** Participants’ hierarchical multiple regression analysis results.

Predictor	*B*	S. E.	*β*	*t*	*p*
Step 1					
BMI	−0.14	0.25	−0.02	−0.56	0.58
Gender	−0.25	1.49	−0.01	−0.17	0.87
Academic year	1.02	0.69	0.06	1.49	0.14
Department	−2.79	1.00	−0.11	−2.78	0.01*
Type of teaching	−3.62	1.54	−0.09	−2.35	0.02*
Step 2					
Physical activity barriers	4.53	0.37	0.45	12.10	<0.001***
Model statistics					
Model	*R*	*R* ^2^	Δ*R*^2^	*F*	*p*
Step 1	0.129	0.017	---	2.40	0.049*
Step 2	0.479	0.230	0.213	27.96	<0.001***

*p < 0.05, **p < 0.01, ***p < 0.001, *R*^2^ = R-squared, Δ*R*^2^ = *R*^2^ ahange, B = Estiamte, *β* = Stand. Estiamte, S. E. = Standart Error.

After controlling for demographic variables, a hierarchical multiple regression analysis was conducted to examine whether barriers to physical activity predicted nomophobia. First, the model included demographic variables such as body mass index, gender, grade level, department and type of education. In the second step, physical activity barriers were added to the model. The final regression model was statistically significant [*R* = 0.479, *R*^2^ = 0.23, adjusted *R*^2^ = 0.22, *F* (6, 563) = 27.955, *p* < 0.001], showing that the estimators explained 23% of the variance in nomophobia. Comparing the hierarchical models showed that adding physical activity barriers significantly increased the model’s explanatory power (Δ*R*^2^ = 0.213, F change = 77.77, *p* < 0.001). This indicates that physical activity barriers contribute significantly to predicting nomophobia beyond demographic variables. Physical activity barriers were the strongest predictor of nomophobia (*β* = 0.451, *p* < 0.001). Regarding individual predictors, the strongest predictor of nomophobia was physical activity barriers (*β* = 0.451, *t* = 12.10, *p* < 0.001). Additionally, department (*β* = −0.106, *p* = 0.006) and type of teaching (*β* = −0.089, *p* = 0.019) were also found to be significant predictors. However, BMI (*p* = 0.579), gender (*p* = 0.867) and academic year (*p* = 0.138) were not significant predictors of nomophobia.

## 4 Discussion

This study examined the predictive role of physical activity barriers in relation to nomophobia, while controlling for various demographic variables. Hierarchical regression analysis revealed that physical activity barriers are a significant and robust predictor of nomophobia. Specifically, physical activity barriers accounted for a substantial proportion of the variance in nomophobia beyond that explained by demographic characteristics.

A study of individuals aged over 20 in Türkiye found that more than 70% of participants experienced nomophobia (ranging from low to high), with increased levels of nomophobia being associated with higher daily smartphone usage and the presence of multiple smartphones in the household (44). Nomophobia is also known to have negative behavioral, physical, and psychological effects, particularly among adolescents and young adults (45). Studies conducted on undergraduate students have shown that increased nomophobia is associated with decreased physical activity (46, 47), and conversely, increased physical activity is associated with decreased nomophobia (48, 49). The findings revealed that barriers to physical activity were the strongest predictor of nomophobia (*β* = 0.451). This suggests that individuals who encounter more barriers to physical activity tend to report higher levels of nomophobia. One possible explanation for this is that people who face such barriers may engage in more sedentary behaviors, such as smartphone use, which could lead to increased dependence on mobile devices. Therefore, the current findings support the idea that limited participation in physical activity is associated with nomophobia.

Another important finding of the study is that the department and type of education are significant determinants of nomophobia. A review of the literature reveals that nomophobia levels and related factors have been examined among undergraduate students in various departments, particularly health sciences students (50, 51), as well as students in other departments (52, 53). However, one study found that young athletes, like the general young population, also exhibit interrelated levels of fear of missing out, nomophobia, and internet addiction (54). These results suggest that the academic environment and educational structure may influence students’ smartphone usage habits and dependence on mobile devices. Differences in academic workload, learning environments, and digital learning applications may partly explain this relationship.

However, BMI, gender and social class were not found to be significant predictors of nomophobia. Given the multitude of factors that can influence BMI (e.g., health status, nutrition and physical inactivity), it is not surprising that no association was found between BMI and nomophobia. A recent study classified nomophobia-related factors as demographic characteristics (e.g., gender and age), smartphone use, lifestyle and academic, psychosocial and clinical variables. The lifestyle factor included in this study encompassed daily living habits such as physical activity, BMI, eating habits, sleep, and smoking. Although fewer studies have been conducted on physical activity, this area has the highest number of studies and percentage of relationships between them (55). A cross-sectional study of young adults (aged 18–35) revealed an association between nomophobia and insomnia, but not with BMI (56). Similarly, a study of nursing students in Turkey found no significant relationship between nomophobia levels and body mass index (57). A large-scale study of 4,317 university students in three countries (mainland China, Taiwan, and Malaysia) reported that those experiencing nomophobia or weight-related self-stigma were more likely to increase their physical activity (58). This is an interesting result in terms of the possibility of a negative situation contributing to a positive outcome, which is contrary to what might be expected. These findings suggest that nomophobia may be more strongly associated with behavioral and lifestyle factors than with basic demographic characteristics. This is consistent with previous studies showing that psychological and behavioral variables are more important in predicting problematic smartphone use.

A review of the literature reveals that only a limited number of studies have examined sports science faculty students, and none of these have investigated the predictive potential of perceived barriers to physical activity in relation to nomophobia. One of the few existing studies examined the relationship between nomophobia and physical activity levels among sports science students in Türkiye. No statistically significant relationship was found between students’ nomophobia levels and their level of physical activity (59). While some studies suggest that individuals who engage in sports have lower levels of nomophobia than those who do not (60, 61), others claim that sports have no effect on nomophobia (62). Overall, the findings emphasize the importance of encouraging university students to participate in physical activity. Providing more opportunities for physical activity and reducing barriers can encourage healthier lifestyle behaviors and reduce excessive smartphone use. Therefore, universities and public health experts should consider developing interventions that reduce barriers to physical activity and promote active lifestyles, thereby addressing technology-related behavioral issues such as nomophobia.

### 4.1 Conclusion

This study investigated the predictive role of physical activity barriers in nomophobia among university students using a hierarchical multiple regression approach. The findings revealed that physical activity barriers remained a significant predictor of nomophobia even when demographic variables were considered. Specifically, physical activity barriers were found to contribute significantly to explaining the variance in nomophobia, indicating that individuals who perceive greater barriers to participating in physical activity tend to report higher levels of nomophobia. Furthermore, department and type of education were identified as significant predictors, suggesting that certain academic factors may influence smartphone addiction among students. However, demographic variables such as BMI, gender, and grade level were not found to significantly predict nomophobia.

### 4.2 Limitations

Despite the important findings of this study, several limitations should be acknowledged. Firstly, the cross-sectional design means that the study is unable to establish a causal relationship between physical activity barriers and nomophobia. Future studies employing longitudinal or experimental designs could provide clearer insight into the underlying mechanisms of this relationship. Secondly, data were collected using self-reported questionnaires. Self-reported measures may be subject to response bias, social desirability bias or inaccurate self-perception. Future research could incorporate objective measures of physical activity, such as wearable activity trackers, to obtain more precise data. Thirdly, the sample consisted of university students from one specific population, which limits the generalizability of the findings to other age groups or cultural contexts. Further research involving different populations and cultural backgrounds is required to validate the robustness of the results. Finally, while the model explains a significant proportion of the variance in nomophobia, other psychological or behavioral factors (e.g., digital addiction, loneliness, or stress) may also play an important role and should be considered in future research.

### 4.3 Theoretical and practical implications

This study makes several important contributions to existing literature on this topic. Firstly, while previous studies have examined the relationship between physical activity levels and nomophobia, this study broadens the scope of this research area by investigating perceived barriers to physical activity as predictors of nomophobia. Thus, it provides a more comprehensive understanding of how lifestyle barriers may influence technology-related behavior patterns among university students. Secondly, the study contributes to the literature by focusing on students enrolled in sports science faculties, a population that has received relatively little attention in nomophobia research. As these students are expected to lead active lifestyles due to their academic training, investigating nomophobia in this group provides valuable insights into the behavioral patterns of young adults closely involved in sports and physical activity. Thirdly, the findings emphasize the potential impact of perceived barriers to physical activity on sedentary behavior and digital technology usage. From a theoretical perspective, the results imply that barriers to active behavior may indirectly lead to increased smartphone dependency. This relationship supports a broader framework linking sedentary lifestyles to problematic technology use and behavioral addictions. From a practical standpoint, the results suggest that interventions aimed at alleviating nomophobia among university students could be improved by addressing perceived barriers to physical activity. Universities and other educational institutions may therefore wish to consider developing strategies that promote active lifestyles and reduce structural and psychological barriers to participation in physical activity. This could involve providing accessible sports facilities, promoting campus-based physical activity programs, and raising awareness of the importance of maintaining a balanced relationship between digital technology use and active living.

## Data Availability

The raw data supporting the conclusions of this article will be made available by the authors, without undue reservation.
