# Supplementary material for: Perceived physical activity barriers as predictors of nomophobia levels in sports science students
Source: Front Public Health. 2026 Mar 30;14:1726076. doi: 10.3389/fpubh.2026.1726076 (PMC13070901; doi:10.3389/fpubh.2026.1726076)
Supplement: Supplementary file 1 [file Data_sheet.docx]

Supplementary Material

# Supplementary Figures and Tables

## Supplementary Figures

Supplementary Figure 1. Conceptual model of the relationship between perceived physical activity barriers and Nomophobia


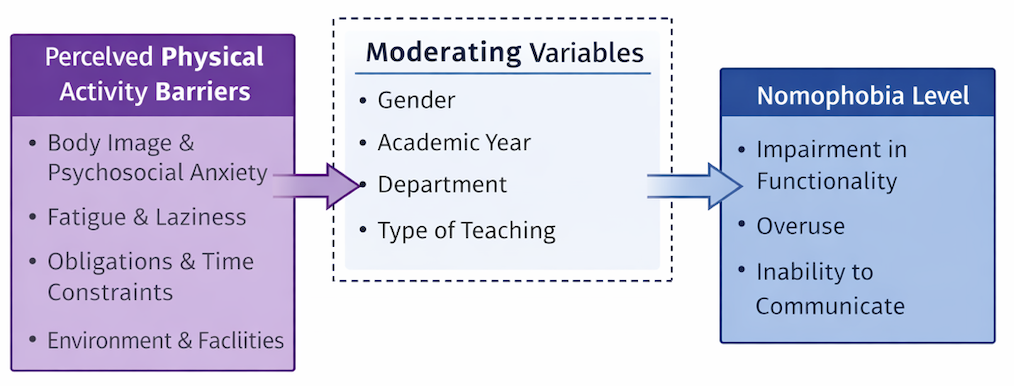


## Supplemantary Tables

**Table 1** Demographic characteristics of the participants

| **Variables** | **Group** | **f** | **%** |
| --- | --- | --- | --- |
| **Grade** | *1.Grade* | 112 | 19.7 |
|  | *2.Grade* | 228 | 40.0 |
|  | *3.Grade* | 122 | 21.4 |
|  | *4.Grade* | 108 | 18.9 |
| **Department** | *Coaching* | 289 | 50.7 |
|  | *Sport Management* | 191 | 33.5 |
|  | *Physical Education* | 90 | 15.8 |
| **Teaching Type** | *Normal Teaching* | 417 | 73.2 |
|  | *Night Teaching (after 5pm)* | 153 | 26.8 |
| **Nomophobia** | *Nope* | 216 | 37.9 |
|  | *Low Level* | 267 | 46.8 |
|  | *Mid- Level* | 83 | 14.6 |
|  | *High Level* | 4 | 0.7 |

**Table 2** Mean and normality analysis results of the data

| **Scales** | **Sub-Dimensions** | **N** | **X̄** | **Sd** | **Skewness** | **Kurtosis** | **Cronbach's α** |
| --- | --- | --- | --- | --- | --- | --- | --- |
| **Nomophobia** | *Impairment in Functionality* | 570 | 19.3 | 7.4 | 0.7 | -0.2 | 0.89 |
|  | *Overuse* | 570 | 19.8 | 7.1 | 0.3 | -0.3 | 0.89 |
|  | *Inability to Communicate* | 570 | 16.8 | 6.5 | 0.3 | -0.5 | 0.89 |
|  | *Total Scale* | 570 | 55.9 | 17.9 | 0.2 | -0.4 | 0.94 |
| **Physical Activity Barriers** | *Body image and Psychosocial anxiety* | 570 | 2.7 | 0.4 | 0.5 | -0.7 | 0.86 |
|  | *Fatigue and Laziness* | 570 | 3.7 | 0.3 | 0.1 | -0.4 | 0.78 |
|  | *Obligations and Time Constraints* | 570 | 3.6 | 0.7 | 0.2 | -0.8 | 0.65 |
|  | *Environment and Facilities* | 570 | 3.2 | 0.8 | 0.2 | -0.9 | 0.74 |
|  | *Total Scale* | 570 | 3.3 | 0.1 | 0.3 | -0.2 | 0.91 |

**Table 3** Correlation results for Body Mass Index, nomophobia and physical activity barriers scores

| **Variables** | **Body Mass Index** | **Nomophobia** | **Physical Activity Barriers** |
| --- | --- | --- | --- |
| **Body Mass Index** | 1.0 |  |  |
| **Nomophobia** | 0.01 | 1.0 |  |
| **Physical Activity Barriers** | 0.07 | **0.46***** | 1.0 |
| *: p<0.05 | | | |

**Table 4** Participants' Hierarchical Multiple Regression Analysis Results

| **Predictor** | **B** | **S.E.** | **β** | **t** | **p** |
| --- | --- | --- | --- | --- | --- |
| **Step 1** |  | | | | |
| ***BMI*** | -0.14 | 0.25 | -0.02 | -0.56 | 0.58 |
| ***Gender*** | -0.25 | 1.49 | -0.01 | -0.17 | 0.87 |
| ***Academic Year*** | 1.02 | 0.69 | 0.06 | 1.49 | 0.14 |
| ***Department*** | -2.79 | 1.00 | -0.11 | -2.78 | 0.01* |
| ***Type of Teaching*** | -3.62 | 1.54 | -0.09 | -2.35 | 0.02* |
| **Step 2** |  | | | | |
| ***Physical Activity Barriers*** | 4.53 | 0.37 | 0.45 | 12.10 | <0.001*** |
| **Model Statistics** |  |  |  |  |  |
| **Model** | **R** | **R^2^** | **ΔR^2^** | **F** | **p** |
| ***Step 1*** | 0.129 | 0.017 | --- | 2.40 | 0.049* |
| ***Step 2*** | 0.479 | 0.230 | 0.213 | 27.96 | <0.001*** |

**p<0.05, **p<0.01, ***p<0.001, R^2^=R-squared,* ***Δ****R^2^=R^2^ ahange, B=Estiamte, β=Stand.Estiamte, S.E.=Standart Error*
